# Rapid and Sensitive Detection of *Bartonella bacilliformis* in Experimentally Infected Sand Flies by Loop-Mediated Isothermal Amplification (LAMP) of the Pap31 Gene

**DOI:** 10.1371/journal.pntd.0003342

**Published:** 2014-12-18

**Authors:** Nasikarn Angkasekwinai, Erin H. Atkins, Richard N. Johnson, John P. Grieco, Wei Mei Ching, Chien Chung Chao

**Affiliations:** 1 Uniformed Services University of the Health Sciences, Bethesda, Maryland, United States of America; 2 Naval Medical Research Center, Silver Spring, Maryland, United States of America; 3 Faculty of Medicine Siriraj Hospital, Mahidol University, Bangkok, Thailand; Institut Pasteur, France

## Abstract

**Background:**

Carrion' disease, caused by *Bartonella bacilliformis*, remains truly neglected due to its focal geographical nature. A wide spectrum of clinical manifestations, including asymptomatic bacteremia, and lack of a sensitive diagnostic test can potentially lead to a spread of the disease into non-endemic regions where competent sand fly vectors may be present. A reliable test capable of detecting *B. bacilliformis* is urgently needed. Our objective is to develop a loop-mediated isothermal amplification (LAMP) assay targeting the pap31 gene to detect *B. bacilliformis.*

**Methods and Findings:**

The sensitivity of the LAMP was evaluated in comparison to qPCR using plasmid DNA containing the target gene and genomic DNA in the absence and presence of human or sand fly DNA. The detection limit of LAMP was 1 to 10 copies/µL, depending on the sample metrics. No cross-reaction was observed when testing against a panel of various closely related bacteria. The utility of the LAMP was further compared to qPCR by the examination of 74 *Lutzomyia longipalpis* sand flies artificially fed on blood spiked with *B. bacilliformis* and harvested at days (D) 1, 3, 5, 7 and 9 post feeding. Only 86% of sand flies at D1 and 63% of flies at D3 were positive by qPCR. LAMP was able to detect *B. bacilliformis* in all those flies confirmed positive by qPCR. However, none of the flies after D3 were positive by either LAMP or qPCR. In addition to demonstrating the sensitivity of the LAMP assay, these results suggest that *B. bacilliformis* cannot propagate in artificially fed *L. longipalpis*.

**Conclusions:**

The LAMP assay is as sensitive as qPCR for the detection of *B. bacilliformis* and could be useful to support diagnosis of patients in low-resource settings and also to identify *B. bacilliformis* in the sand fly vector.

## Author Summary

Carrion's disease, caused by *Bartonella bacilliformis* remains truly neglected due to its focal geographical nature. A wide spectrum of clinical manifestations, including asymptomatic bacteremia can potentially lead to a spread of the disease into non-endemic regions. The PCR-based approach is sensitive for detection of *B. bacilliformis* but requires a thermocycler, thus limiting its use in remote endemic areas. LAMP is a simple method capable of detecting *B. bacilliformis* DNA within an hour under isothermal conditions, requiring less specialized equipment for amplification, thus enabling diagnosis in rural areas. This study demonstrated that the detection limit of LAMP, targeting the pap31 gene of *B. bacilliformis*, was comparable to that of qPCR. With a high, targeted selectivity, LAMP showed a high specificity as no cross-reaction was observed when testing a panel of closely related bacteria. The utility of the LAMP assay was further demonstrated by the examination of sand flies artificially fed on blood spiked with *B. bacilliformis*. The results showed that LAMP was able to detect *B. bacilliformis* in all flies confirmed positive by qPCR. This study showed that LAMP can be useful to support diagnosis of patients in low-resource settings and also to identify *B. bacilliformis* in the sand fly vector.

## Introduction

Carrion's disease, caused by *Bartonella bacilliformis* remains truly neglected due to its focal geographical nature, occurring in small rural communities of inter-Andean valleys between altitudes of 500–200 meters above sea level in Peru, Columbia and Ecuador [1]. The disease typically presents with acute fever and devastating hemolysis known as Oroya fever, followed by asymptomatic bacteremia and chronic eruptive disease, called Verruga peruana [2]. As the period of asymptomatic bacteremia may vary and last for up to 15 months, these individuals can presumably serve as a reservoir for the bacteria, leading to potential transmission by the putative sand fly vector, *Lutzomyia verrucarum*, to either populations living in endemic areas or travelers visiting such regions [3]. The increase of human migration, habitat destruction, as well as adaptation of sand flies association with human, may also aid the spread of the potential vector [4]. With changes in weather patterns or host dynamics, sporadic and occasional epidemics of bartonellosis have occurred over a much larger area than previously described [5]–[8].

*L. verrucarum* have been found throughout the western half of Peru between 1500 and 3200 meters above sea level in Occidental and Interandean valleys of the Andes mountain [9]. This species was presumed to be the primary vector of *B. bacilliformis*, based on the evidence derived from the presence of the flies in the areas in which the disease occurred [8]. However, Carrion's disease is found in areas where *L. verrucarum* is not present which implied that other *Lutzomyia* sand flies may serve as a vector [4]. Additionally, the mechanisms by which transmission occurs, mechanically through surface contamination of the sand fly or biologically through *B. bacilliformis* infected sand flies, remain unanswered.

With a recent increase of migration in human populations and the possible existence of other unrecognized vector species as well as a great abundance of other sand flies species such as *Lutzomyia longipalpis* at the lower elevations in South America, a growing concern is whether or not other sand flies species are susceptible to *B. bacilliformis* and have the capability of transmitting infection. Generally, oviposition of sand flies starts on the fifth day after blood meal. If sand flies were able to transmit *B. bacilliformis*, the bacteria should be able to establish infection in sand flies through this period and persist long enough to be transmitted [10].

Diagnosis of bartonellosis remains challenging because each test has its own limitations. Gimsa-stained blood smear is the cheapest and quickest diagnostic method being used to diagnose acute disease but the test suffers from low sensitivity (36%) [11]. Serological tests such as IFA or immunoblot show promising results but require paired samples of acute and convalescent phases and may not be useful to diagnose acute disease [12]. Isolation of the bacteria may require up to 4 weeks before they are considered negative [13], thus it may not be useful for case management. PCR amplification of DNA shows promising sensitivity and specificity but requires a high-precision thermal cycler, thus it is impractical for diagnosing the disease in remote areas [11]. A rapid and sensitive test capable of detecting *B. bacilliformis* DNA is of great clinical importance, not only for early diagnosis and treatment but for better understanding of true disease burden, natural history of the disease as well as the role of *L. verrucarum* or other *Lutzomyia* species in transmission of the disease.

Loop-mediated isothermal amplification (LAMP) is a nucleic acid amplification method, generating up to 10^9^ fold amplification within an hour under isothermal conditions; hence, it is simpler and requires less specialized equipment than conventional or real time PCR [14]. In this study, we aimed to develop the LAMP assay targeting heme-binding protein pap 31 gene for detection of *B. bacilliformis* compared to qPCR using known positive samples including plasmid containing the targeted gene and *B. bacilliformis* genomic DNA. Additionally, the utility of the LAMP assay was evaluated by testing *L. longipalpis* fed on blood infected with *B. bacilliformis* and comparing the detection limit to that of qPCR. A recent study has shown that an *in vitro* feeding method using a natural skin membrane and an artificial feeder is a viable alternative to the use of the *in vivo* method using anesthetized hamsters for blood-feeding sand flies [15]. In addition, in the situation where there is no known non-human host like for human bartonellosis, an artificial feeding system is best fitted for studying transmission. According to the results obtained, we should be able to determine whether *B. bacilliformis* would be able to propagate in *L. longipalpis* sand flies.

## Materials and Methods

### Ethics statement

This study was reviewed and approved by the ethics committee of the Uniformed Services University of the Health Sciences. Human blood used in this study was obtained from stored blood that expired from Armed Services Blood Program (ASBP), Maryland. There was no IACUC review the animal care in this study since there was no animal used other than skin from dead quails which were residual from toxicology testing at U.S. Army Public Health Command (USAPHC).

### Positive control plasmid DNA

To determine the analytical sensitivity of the LAMP assay, a plasmid containing the pap31 gene sequence of ATCC strain 35685 (KC 583) (accession number DQ207957) of *B. bacilliformis* was constructed as previously described [16]. In brief, the sequence between the forward primer (5′- gcagcatatgttatgatcccgcaagaaata-3′) and the reverse primer (5′-ctaaaggcacaaccacaacgcattcttaag-3′) was amplified. The amplified product was then cloned into pET24a (Novagen, San Diego, CA) and sequence confirmed to be used as the standard for nucleic acid amplification.

### Bacteria strains

The fluorescent strain of *B. bacilliformis* KC583 (ATCC 35685) (glo*Bart*) was grown in heart infusion agar blood plates (HIAB; heart infusion agar [Difco, Detroit, Mich.] containing 4% sheep red blood cell [Quad 5, Ryegate, Montana] and 2% sheep serum) using the standard methods described by Minnick for cultivation of *B. quintana* [17], [18].

### Infecting laboratory-reared *L. longipalpis* sand flies with *B. bacilliformis*

Two-to-four day-old colony-reared female sand flies were placed in a 1-pint paper feeding carton fitted with a screen top. All flies were fed through a quail-skin membrane (US Army Public Health Command, Aberdeen Proving Ground, MD) on human blood inoculated with a fluorescent strain of *B. bacilliformis* (glo*Bart*). A membrane feeder fitted with the quail skin membrane was placed on top of the feeding cup with the membrane pressed against the screen. Heparinized human blood inoculated for an hour with fluorescent strain of *B. bacilliformis* (glo*Bart*) was added, using a sterile glass pipette inserted through the top of the feeder into the inner chamber until it was approximately two-thirds full with blood. The flies were allowed to feed under conditions of 22–24°C and 75–80% relative humidity. The outer chamber of the membrane feeder was connected to a circulating water bath to maintain the blood temperature at 37°C throughout the feeding period. Fully engorged sand flies were retained in the cages for another 24 h before being transferred to fresh cages. They were kept under the same conditions and were provided a solution of 15% sucrose before being harvested at days (D) 1, 3, 5, 7, and 9 post blood feeding. At least 10 fully engorged females were harvested at each day.

### DNA extraction

DNA extraction of the whole genome of *B. bacilliformis* was performed using the DNeasy Blood & Tissue Kit (QIAGEN, Germany) following the manufacture's protocol. To extract DNA from non-infected sand flies spiked with *B. bacilliformis*, 20 µL of a culture suspension of *B. bacilliformis* was added to 180 µL of Buffer ATL (QIAGEN, Germany) and a single sand fly was added to the solution. Similarly, 180 µL of Buffer ATL was added to single or pooled, artificially-fed sand flies before being homogenized with a disposable plastic homogenizer. The mixture was added together with 20 µL of proteinase K and incubated at 56°C for 2 h. Next, 200 µL of Buffer AL (QIAGEN, Germany) was added to the sample and incubated at 70°C for 10 min. Then, the DNA extraction was performed using the manufacture's protocol. Two elutions were done with 50 µL of low EDTA buffer (final volume was 100 µL) and each time the buffer was incubated on the column for 10 min.

### LAMP primer design and LAMP reaction

The oligonucleotide primers used for the LAMP assay were designed based on the pap31 gene sequence from the ATCC KC 583 strain of *B. bacilliformis*. Using PREMIER Biosoft (http://premierbiosoft.com), a highly specific set of primers including two outer (F3 and B3), two inner (FIP and BIP) and loop primers (LF and LB) were used. All primers were synthesized by Eurofins MWG Operon (Huntsville, AL) and are described in Table 1.

**Table 1:** Description of LAMP and qPCR primers used for detection of *B. bacilliformis.*

Primer name	Length (bp)	Tm (°C)	Sequences 5′- 3′
LAMP primers#2			
-F3	22	58.9	GTTTGGGTTGATAAGGAAGGTA
-B3	21	58.7	TGAACCAATAGCTTGTACCTG
-FIP	38	75.5	GGCCCTTAGCGACCTCAATCCAGGGCAGAGACTCAAGA
-BIP	40	72.9	AAAGTGGGCTGGTGCCACACATAAGGCATAATACGGTCAG
-LF	21	76.8	AATCTGCTTGAAAGCATCTGC
-LB	19	71.8	GTACGCATCGGTTTTGGTG
qPCR primers#1			
-Forward	20	59.5	AGGTGGTTTGTACGCAGGTT
-Reverse	20	59.1	TCTTGAGTCTCTGCCCTGTG

The LAMP reactions were carried out in a 25 µL reaction volume containing 1.6 µM of each of the FIP and BIP primers, 0.4 µM of the F3 and B3 primers, 0.8 µM of the LF and LB primers, 20 mM Tris-HCl (pH 8.8), 10 mM KCl, 8 mM MgSO_4_, 10 mM (NH)_4_SO_4_, 0.1% Triton X-100, 0.8 M betaine (Sigma-Aldrich, St Louis, MO), 1.4 mM dNTP mixture (New England Biolabs, Beverly, MA), 8 U Bst DNA polymerase (New England Biolabs, Beverly, MA), and 5 µL of DNA template. All reactions were prepared in duplicate. The reaction mixture was incubated in a Biometra thermocycler (Applied Biosystems, Foster City, CA) at 59°C for 60 minute. Each reaction was terminated by adding 5 µL of 10X BlueJuice (Invitrogen, Carlsbad, CA). The LAMP products were examined by electrophoresis on a 2% agarose gel stained with a 1∶20,000 dilution of ethidium bromide (Sigma-Aldrich Products, St Louis, MO) and the positive samples were identified by the appearance of a typical ladder banding pattern. LAMP was considered positive when both duplicates were positive.

### Real-time PCR assay

This assay used primers designed against the pap31 gene sequence of the ATCC KC 583 strain of *B. bacilliformis* (https://www.genscript.com/ssl-bin/app/primer). The primer sequences are described in Table 1. In brief, each reaction mixture contained 750 nM of the forward primer, 750 nM of the reverse primer, 1×RT_2_ SYBR Green qPCR Mastermix (SA-Biosciences, Frederick, MD), 5 µL of plasmid or genomic DNA of *B. bacilliformis* or 1 µL of DNA consisting of human or sand fly DNA spiked with *B. bacilliformis* or from that of artificially-fed sand flies and water added to a final volume of 20 µL. The qPCR reactions were performed and analyzed using the 7500 Fast Real-time PCR system (Applied Biosystems, Foster City, CA), with an initial 5 minute activation step at 95°C, followed by 40 cycles of 95°C for 10 seconds, 60°C for 30 seconds and a melting curve determination cycle. The samples were tested in duplicate. The results were reported as mean of Ct values and a detection range expressed in copies/µL.

### Analytical sensitivity and specificity of LAMP primers

To determine the analytical sensitivity, 10-fold serial dilutions of plasmid DNA across a range of 106 to 1 copy (per reaction) and sterile water were used to define the limit of detection (LOD) as compared to qPCR. When using genomic DNA of *B. bacilliformis* as the template, the copy number was determined by qPCR and the LOD of LAMP was determined as compared to that of qPCR. In addition, to ensure that various host components would not interfere with the detection limit of the LAMP assay, the genomic DNA of *B. bacilliformis* was spiked with normal human plasma and a single clean sand fly before extraction.

The analytical specificity of LAMP was determined using genomic DNA of various *Rickettsia* species including *R. conorii, R. rickettsii, R. typhi*, *Orientia tsutsugamushi, Leptospira interrogans*, *B. henselae*, the species of bartonella that are abundant in Peru including *B. quintana* and *B. rochalimae* (ATCC BAA-1498) as well as *Leishmania* spp. that are present in Peru including *L. mexicana* and *L. braziliensis*. Furthermore, interference studies were done by spiking the various bacteria or protozoa (100 copies per reaction) with the genomic or plasmid DNA of *B. bacilliformis* (10 copies per reaction). Based on BLAST search using pap31 against the entire *B. bacilliformis* genomic DNA, the number of organisms (i.e. number of copies) is equivalent to the number of pap31 gene.

### Detection of *B. bacilliformis* in experimentally infected sand flies

The utility of the LAMP was further compared to qPCR by examining 74 laboratory reared colonies of *L. longipalpis* female sand flies that fed on *B. bacilliformis* infected blood. Sensitivity was calculated as (number of true positive)/(number of true positives + number of false negatives), and specificity was calculated as (number of true negatives)/(number of true negatives+ number of false positives).

## Results

### Analytical sensitivity and specificity of LAMP assay

The detection limit of the LAMP assay compared to qPCR against different types of known positive samples for *B. bacilliformis* is shown in Table 2. The LAMP assay was as sensitive as qPCR for the detection of *B. bacilliformis*, with a detection limit of 1 to 10 copies/µL, depending on the sample metrics.

**Table 2:** Detection limit of the LAMP assay compared to qPCR targeting different types of known positive samples for *B. bacilliformis.*

Type of samples	qPCR, copies/µL^@^ (range)	Mean Ct value	LAMP, copies/µL
Plasmid	2	39.1	2
Genomic DNA	3.6 (1, 7)	38.8	1
Genomic DNA in presence of human DNA	10 (8, 12)	39.4	10
Genomic DNA in presence of sand fly DNA	18 (13, 23)	38.6	8

@ Mean copies number.

**Figure 1:**
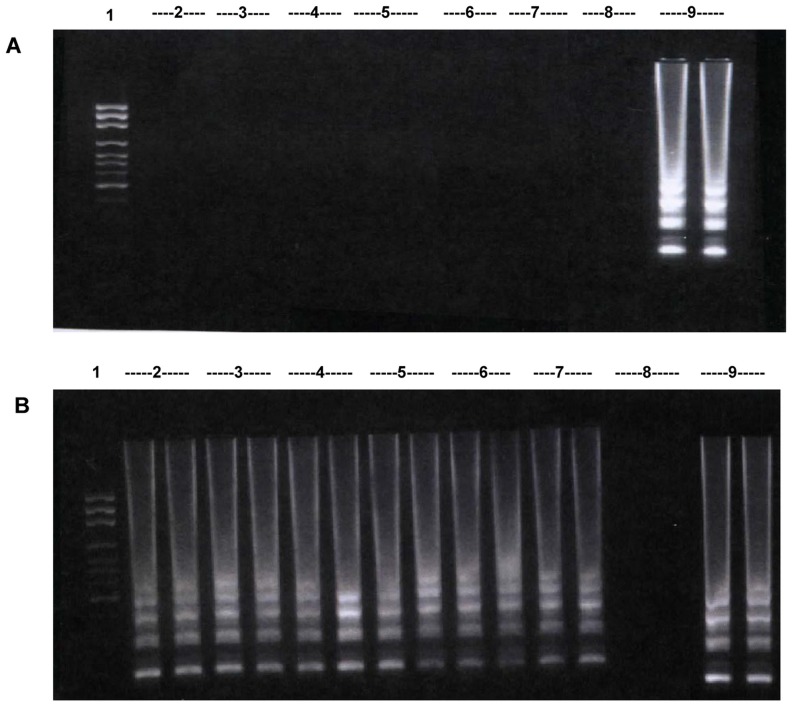
Demonstration of analytical specificity of pap 31 LAMP. **A.** Lane 1: 100 bp ladder, Lane 2–7: products from LAMP reactions containing genomic DNA from *R. conorii, R. rickettsii, R. typhi, Orientia tsutsugamushi, L. interrogans, and B. henselae*, respectively. Lane 8: negative control, Lane 9: positive control. **B.** LAMP assay performed using a combination of *B. bacilliformis* genomic DNA (10 copies/reaction) and other bacterial genomic DNA(100 copies/reaction). Lane 1: 100 bp ladder, Lane 2–6, 9: *B. bacilliformis* plus either *R. conorii*, or *R. rickettsii*, or *R. typhi*, or *Orientia tsutsugamushi*, or *L. interrogans*, or *B. henselae*, respectively. Lane 7: positive control, Lane 8: negative control.

When testing the LAMP assay using DNA from closely related bacteria including various *Rickettsia* spp. (n = 3), *Orientia tsutsugamushi* (n = 1), *L. interrogans* (n = 1) as well as *B. henselae* (n = 1) as the template, no amplification was observed for any of these bacteria (Fig. 1A). The LAMP assay developed here did not detect *B. henselae* even though both possess pap31 gene. Furthermore, no interference of amplification was observed when testing the LAMP assay with the samples containing genomic DNA of *B. bacilliformis* and that of other bacteria (Fig. 1B). When testing LAMP assay using only genomic DNA of *B. quintana* (n = 1) or *B. rochalimae* (n = 1) and two species of *Leishmania* including *L. mexicana* (n = 1) and *L. braziliensis* (n = 1) at 5,000 copies per reaction (i.e. 500 fold more than the detection limit of *B. bacillformis* [10 copies per reaction]), no amplification was observed for any of these organisms. Additionally, the presence of 10 fold excess of *B. quintana*, *B. rochalimae* or two species of *Leishmania* DNA to *B. bacilliformis* plasmid DNA showed that there were no interferences on amplification of *B. bacilliformis* DNA by pap31 LAMP assay. Furthermore, there was no cross reactivity between *B. bacilliformis* primers and DNA extracted from human blood.

### Examination of sand flies fed on blood infected with *B. bacilliformis*

Laboratory reared colonies of *L. longipalpis* female sand flies (n = 74) were fed through a quail skin membrane on blood containing *B. bacilliformis*. The number of blood-fed flies that were harvested at the day of interest was as follows: D1 (n = 15), D3 (n = 11), D5 (n = 12), D7 (n = 16) and D9 (n = 20). The bacterial DNA was extracted from individual sand flies from D1 and D3. Four individual sand flies from D5, 7 and 9 along with 2 pools (4 flies/pool) from D5, 3 pools (4 flies/pool) from D7 and 4 pools (4 flies/pool) from D9 were extracted. Flow diagram of a diagnostic accuracy in detection of pap31 in sand flies by LAMP compared to qPCR was shown in S1 Figure.

As shown in Table 3, 86% of *L. longipalpis* (13/15) at D1 post feeding on infected blood were positive by qPCR, with the mean copy number of 64.6 copies/µL (range 7–382 copies/µL) and 63% of flies (7/11) at D3 post feeding were positive by qPCR, with the mean copy number of 71 copies/µL (range 10-302 copies/µL). All individual and pooled flies after D3 were negative by qPCR assay.

**Table 3:** Detection of pap31 in *L. longipalpis* sand flies from day (D) 1 to D9 post feeding on blood infected with *B. bacilliformis* by qPCR and LAMP.

	D1 (n = 15)D3 (n = 11)D5 (n = 6)D7 (n = 7)D9 (n = 8)	
LAMP*	qPCR										
	(+)	(−)	(+)	(−)	(+)	(−)	(+)	(−)		(+)	(−)
Positive (+)	13	2	7	1	0	2	0	0		0	0
Negative (-)	0	0	0	3	0	4	0	7		0	8
Total	13	2	7	4	0	6	0	7		0	8
Percentage with											
positive qPCR#	86% (13/15)		63% (7/11)		0% (0/6)		0% (0/7)			0% (0/8)	

A total of 12 sand flies at D5 includes 4-individual sand flies plus 2 pools (4 SF/pool) (No. of samples  = 6).

A total of 16 sand flies at D7 includes 4-individual sand flies plus 3 pools (4 SF/pool) (No. of samples  = 7).

A total of 20 sand flies at D9 includes 4-individual plus 4 pools (4 SF/pool) (No. of samples  = 8).

*LAMP was considered positive when both duplicates were positive.

# Proportion of samples positive by qPCR from all samples tested on each day.

As shown in Table 4, the sensitivity of the LAMP assay at D1 and D3 was 100%. The specificity of the LAMP assay at D3, D5, D7 and D9 was 75%, 66.6%, 100% and 100%, respectively. Our results suggest that LAMP targeting the pap31 gene is highly sensitive and moderately specific for detection of *B. bacilliformis* in experimentally infected sand flies compared to qPCR.

**Table 4:** Sensitivity and specificity of pap31 LAMP assay in detecting *B. bacilliformis* from experimentally infected sand flies compared to qPCR.

	D1	D3	D5	D7	D9
Sensitivity*	100% (13/13)	100% (7/7)	NA	NA	NA
Specificity**	NA	75% (3/4)	66.6% (4/6)	100% (7/7)	100% (8/8)

* Sensitivity was calculated as (number of true positive)/(number of true positives + number of false negatives)

**Specificity was calculated as (number of true negatives)/(number of true negatives+ number of false positives).

## Discussion

We reported here the development of the LAMP assay targeting the pap31 gene to detect *B. bacilliformis*. The limit of detection (LOD) for the LAMP assay ranged from 1 to 10 copies/µL, depending on the sample metrics, which was comparable to that of real-time PCR which ranged from 2 to 18 copies/µL. In addition, no cross-reaction was observed when testing a panel of other closely related bacteria. We also demonstrated the capability of the pap31 LAMP assay to identify *B. bacilliformis* in the sand fly vector. LAMP was able to detect *B. bacilliformis* in all those flies confirmed positive by qPCR. However, none of the flies after D3 were positive by either LAMP or qPCR.

Carrion's disease caused by *B. bacilliformis*, has been described as an exotic disease, confined to remote areas of certain mountain regions in South America [1]. Once infected, the patients can remain persistently bacteremic due to the existence of the intra-erythrocytic phase, thus providing a protective niche for the bacteria that is competent for vector transmission [19]. The PCR-based approach appears to be a sensitive method of detection of *B. bacilliformis*. Previous studies used PCR methods targeting several genes including the *gltA* gene, the *rpoB* gene, the 16S-23S rRNA ITS, or the *ftsZ* gene in attempts to classify the *Bartonella* species [20]. There have been reports of using PCR for diagnosis of infection caused by *Bartonella* spp. and Carrion's disease [21]–[23]. Although these molecular techniques offer a high sensitivity and specificity, it requires a dedicated thermocycler, thus limiting its use in remote rural endemic area. Alternatively, the LAMP method is rapid and simple to perform, requiring only a water bath or heating block for amplification. A sufficient amount of amplified product can be produced within an hour under isothermal conditions, enabling a rapid, molecular diagnosis in rural areas [14].

In the recent years, several studies have indicated that LAMP has a high sensitivity and specificity compared to either conventional, nested or real time PCR for the detection of several intracellular bacteria, such as *Coxiella burnetii* or *Orientia tsutsugamushi* [24], [25] or in distinguishing species of several intraerythrocytic protozoan parasites, such as *Plasmodium* spp. or *Babesia* spp. [26], [27]. The LAMP assay is also specific due to recognition of six distinct sequences on the targeted gene by six primers. This was demonstrated in our results as no cross-reaction was observed when testing other closely related bacteria. In addition, our targeted gene sequence (pap31) is unique to *B. bacilliformis* which has only 8% identity with other *Bartonella* spp. Since most of the homologous sequences are located in the 3′ end of the DNA and all our primers are located outside of the homologous region, thus the target is unique in detecting *B. bacilliformis*. In addition, neither genomic DNA of human, sand fly nor that of closely related bacteria inhibited DNA amplification in the pap31 LAMP assay.

Previous studies have shown the capability of LAMP for detecting pathogens in mosquitos including *Plasmodium* spp., *Dirofilaria immitis* and *Wuchereria bancrofti* [28]–[30]. This study is the first report whereby LAMP assay was used for detecting *B. bacilliformis* in sand fly vectors. The utility of the pap31 LAMP assay was evaluated by examining artificially-fed *L. longipalpis* sand flies on blood infected with *B. bacilliformis*. When using qPCR as a reference standard, 86% of flies at D1 and 63% of flies at D3 post feeding were positive by qPCR. Overall, LAMP shows a sensitivity of 100% at D1 and D3 and specificity of 100% at D5 and D9 post feeding. However, DNA from one sand fly at D3 and 2 sand flies at D5 was unable to be amplified by qPCR but showed positive for *B. bacilliformis* via the LAMP assay, resulting in lower specificity of the LAMP assay. Regarding the selectivity of the LAMP assay, a false positive reaction of LAMP is less likely. It must be noted that the pap31 LAMP assay has a slightly lower LOD than that of qPCR. It is possible that the number of bacteria in those sand flies is too low to be detected by qPCR but not by LAMP. In addition, the presence of inhibitors such as hemoglobin, in blood fed sand flies may prevent Taq DNA polymerase in the qPCR assay from extending the DNA in the time allowed, adversely affecting the amplification efficiency but do not inhibit Bst DNA polymerase in LAMP assay [14], [31].

*L. longipalpis* is known to be an important vector of American visceral leishmaniasis in Latin America. This sand fly species has been shown to be very permissive of *Leishmania* spp. infection [10]. Even though *Bartonella* spp. has never been isolated from *L. longipalpis*, many bacterial infections have been observed from laboratory reared colonies of *L. longipalpis* [10]. Thus, we used this species to be experimentally infected with *B. bacilliformis*. To date, it remains unclear if *B. bacilliformis* is transmitted through surface contaminations or becomes an established infection in sand flies. If the sand flies can support the bacterial growth through the entire gonotrophic cycle, this would indicate a potential for biological transmission. Therefore, the detection of bacteria in sand flies after five days would support its susceptibility to *B. bacilliformis* infection. The initial pathogen load in the sand flies might affect the detection data for *B. bacilliformis* from D 1–9. However, we are convinced of sufficient pathogen load in the blood that fed on sand fly despite no culture or PCR testing on *B. bacilliformis* spiked blood. Firstly, previous study observed that *B. bacilliformis* had a high erythrocyte invasion rate for up to 80% in patients with Oroya fever which might be owing to a high motility of *B. bacilliformis* as compared to other *Bartonella* spp. [32]. In addition, our prior work (unpublished data), determining the RBC infection rate with *B. bacilliformis* by using flow cytometer showed that the infection rate for all of the blood samples tested (n = 8) was more than 73% (74–89%). Flow cytometry was used since it provided a near real time infection rate which was much more accurate than either culture or PCR testing[33]. Therefore, our results show that none of the flies after D3 were positive by either LAMP or qPCR should suggest that *B. bacilliformis* is incapable of propagating in artificially-fed *L. longipalpis.* While the authors recognize that *L. verrucarum* is the ideal sand fly species to work with, a colony of this species was not available. Future studies should evaluate this technique with *L. verrucarum*.

The ability of pap31 LAMP to detect even a single bacterium of *B. bacilliformis* suggests the capability of this method to identify *B. bacilliformis* in the sand fly vector, which is important for disease control and surveillance. This method could have the potential to support diagnosis of patients in the early stage of illness, thereby leading to prompt initiation of appropriate antimicrobial treatment. With the advantages of being a simple and rapid assay, the LAMP method could be useful for rapid detection of infected sand flies as well as early diagnosis of patients from low-resource settings. Yet, further studies to evaluate this method for the detection of *B. bacilliformis* in suspected patients are needed to further determine the performance of LAMP assay. Future studies should evaluate this assay with samples from patients with different clinical manifestation. Of interest, when this method was used in combination with a sensitive and specific antibody detection such as enzyme-linked immunosorbent assay (ELISA) using recombinant protein Pap31 (rPap31) [31], they will likely improve detection of all truly infected individual and provide a broader window of detection than was possible with either one assay.

## Supporting Information

S1 Figure Flow diagram of a diagnostic accuracy in detection of pap31 in sand flies post feeding on blood infected with *B. bacilliformis* by LAMP compared to qPCR.
(PDF)

S1 Checklist STARD checklist.
(DOC)
