# Supplementary figures and images for: Rapid and Sensitive Detection of Bartonella bacilliformis in Experimentally Infected Sand Flies by Loop-Mediated Isothermal Amplification (LAMP) of the Pap31 Gene
Source: PLoS Negl Trop Dis. 2014 Dec 18;8(12):e3342. doi: 10.1371/journal.pntd.0003342 (PMC4270493; doi:10.1371/journal.pntd.0003342)

# Flow diagram of a diagnostic accuracy of pap-31 LAMP assay compared to qPCR assay

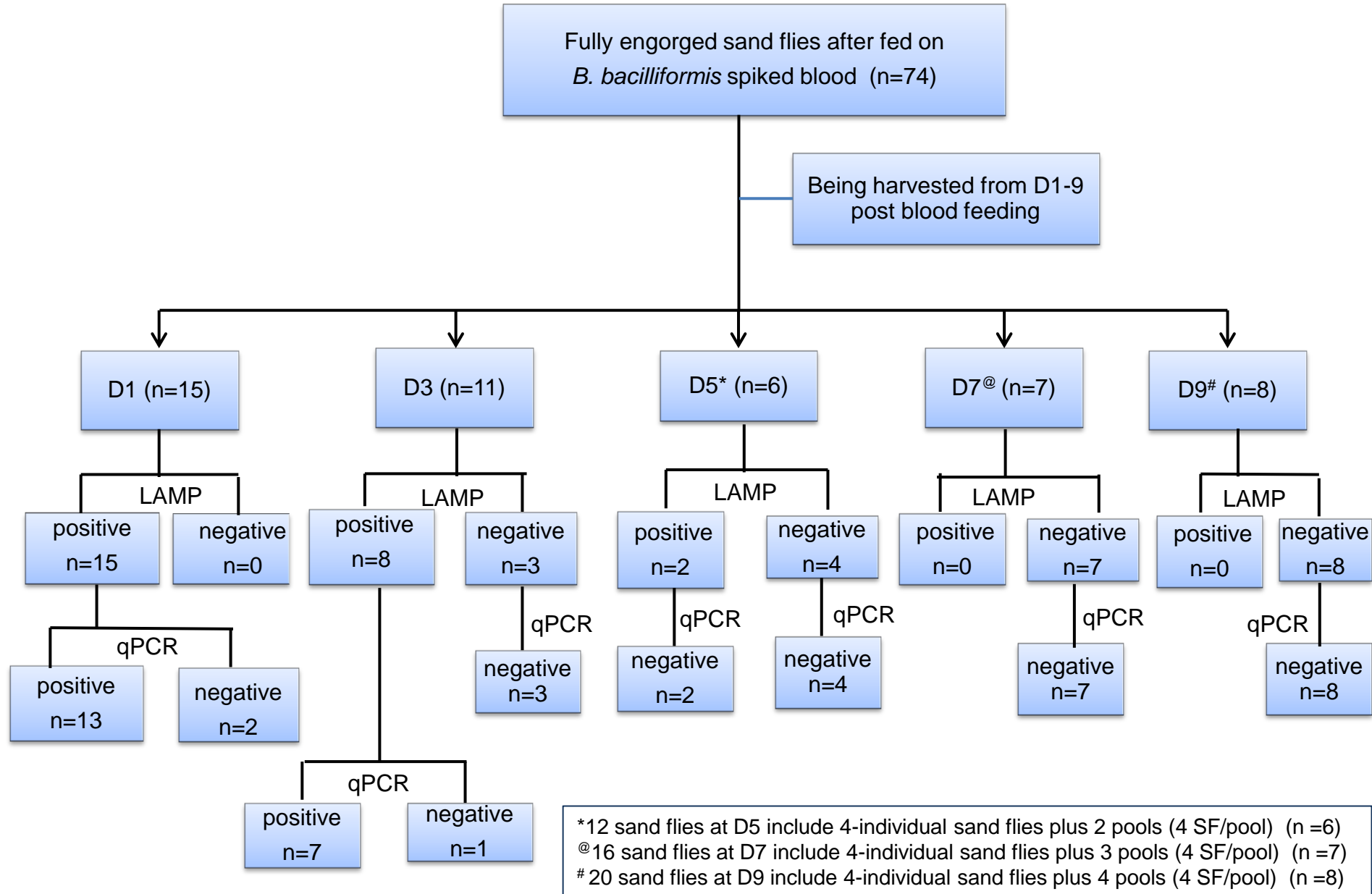

Supplement: S1 Figure — Flow diagram of a diagnostic accuracy in detection of pap31 in sand flies post feeding on blood infected with B. bacilliformis by LAMP compared to qPCR. (PDF) [file pntd.0003342.s001.pdf]
